# A Text-book of Histology and Microscopic Anatomy of the Human Body, Including Microscopic Technique

**Published:** 1903-01

**Authors:** John J. Repp


### BOOK REVIEW.

A Text-book of Histology and Microscopic Anatomy of the Human Body, Including Microscopic Technique. By Dr. Ladislaus Szymonowicz, A. O. Professor of Histology and Embryology in the University of Lemberg. Translated and Edited by John Bruce MacCallum, M.D., Johns Hopkins University, Baltimore. Illustrated with 277 engravings, including 57 plates in color and monochrome. 435 pages. Philadelphia and New York: Lea Brothers & Co., 1902. Cloth, $3.00, net.

A good knowledge of histology is absolutely essential to an adequate conception of such medical subjects as physiology, pathology, therapeutics, and the processes of repair of tissues. Fortunately, there is no branch of medical science which affords a more fascinating study than histology. This is especially so now that Szymonowicz-MacCallum has appeared in the book market. This work is printed in large type upon excellent paper and is attractively and durably bound. The illustrations are noteworthy on account of their abundance and the excellence of their execution. Whenever one feels the need of a figure or plate to explain the text, it is always to be found. Of the plates, some are in color and some in monochrome. These are so inviting as to compel attention to them. They markedly facilitate the study of the subject. Writing as I am for veterinary students and practitioners, it may be well to state that although the title of the book sets forth that it is an "histology of the human body," out of a total of 180 figures and 42 plates, exclusive of those which are diagrammatic, there are 119 figures and 22 plates made from 19 different species of lower animals as against 61 figures and 20 plates made from the human body. The veterinarian, therefore, may study this book with the assurance that it is in reality a safe and helpful guide to him in the study of animal histology. The price is very low, considering the character of the book.

Part I. of the book treats of the *cell* and the various *tissues* which enter into the structure of the animal body. Part II. treats of the *microscopic anatomy* of the various organs. The appendix discusses in a singularly concise and helpful way the subject of *general microscopic technique.* The language of the book is remarkably intelligible and readable, with complete absence of unnecessarily technical language. Due credit is given American authors for their work, something unusual in a foreign book. MacCallum has made numerous additions both in the text and in the illustrations. It is important to note that he has admirably succeeded in embodying the very latest researches upon the subjects with which the book deals. I can heartily and unreservedly recommend this book to students and practitioners of veterinary medicine.—JOHN J. REPP, V.M.D.

